# Epithelial and stromal circadian clocks are inversely regulated by their mechano-matrix environment

**DOI:** 10.1242/jcs.208223

**Published:** 2018-03-01

**Authors:** Jack Williams, Nan Yang, Amber Wood, Egor Zindy, Qing-Jun Meng, Charles H. Streuli

**Affiliations:** Wellcome Centre for Cell-Matrix Research and Manchester Breast Centre, Faculty of Biology, Medicine and Health, University of Manchester, Manchester M13 9PT, UK

**Keywords:** Circadian clocks, Epithelial cells, Fibroblasts, Breast, Lung, Epidermis, Circadian gene expression

## Abstract

The circadian clock is an autonomous molecular feedback loop inside almost every cell in the body. We have shown that the mammary epithelial circadian clock is regulated by the cellular microenvironment. Moreover, a stiff extracellular matrix dampens the oscillations of the epithelial molecular clock. Here, we extend this analysis to other tissues and cell types, and identify an inverse relationship between circadian clocks in epithelia and fibroblasts. Epithelial cells from mammary gland, lung and skin have significantly stronger oscillations of clock genes in soft 3D microenvironments, compared to stiff 2D environments. Fibroblasts isolated from the same tissues show the opposite response, exhibiting stronger oscillations and more prolonged rhythmicity in stiff microenvironments. RNA analysis identified that a subset of mammary epithelial clock genes, and their regulators, are upregulated in 3D microenvironments in soft compared to stiff gels. Furthermore, the same genes are inversely regulated in fibroblasts isolated from the same tissues. Thus, our data reveal for the first time an intrinsic difference in the regulation of circadian genes in epithelia and fibroblasts.

## Abstract (precis)

**Highlighted Article:** Cell-matrix interactions are known to control circadian clocks. Here, we discovered that the matrix inversely regulates epithelia and fibroblasts.

## INTRODUCTION

Most organisms have evolved intrinsic time-keeping mechanisms to entrain their cells to respond to challenges imposed by variations in time. Cell-autonomous circadian clocks regulate patterns of gene expression over 24-h time frames. The body's master clock in the suprachiasmatic nucleus generates robust circadian rhythms that are aligned to day and night cycles, and coordinates the body's time-keeping (Dibner et al., 2010; Reppert and Weaver, 2002; Roenneberg and Merrow, 2005; Takahashi et al., 2008). This clock machinery controls tissue-specific sets of genes – for example in neurons versus glia, pancreatic islet-α versus β cells, atria versus ventricles in the heart, cartilage versus tendon (Guilding et al., 2009; Petrenko et al., 2017; Tong et al., 2013; Yang and Meng, 2016; Yeung et al., 2014). Despite advances in understanding circadian rhythmic outputs, less is known about how these highly conserved molecular clocks adapt to the local niche within peripheral tissues.

In a relatively soft tissue, the mammary gland, several hundred genes are under circadian control (Blakeman et al., 2016). However, the strength of their daily regulation is dependent on the mechano-biological nature of tissue context (Yang et al., 2017). Interestingly, the mechanical extracellular matrix (ECM) microenvironment surrounding epithelial ducts stiffens during ageing, contributing to reduced circadian rhythms in older individuals.

Epithelial organs comprise epithelia and subtending stroma-containing fibroblasts, as well as endothelial, immune and neuronal cells. The epithelia carry out specialist tissue functions, while fibroblasts contribute to the cellular microenvironment consisting of stromal ECM. This ECM impacts both on epithelial cell function and builds overall tissue architecture. Importantly, different cell types can reside in regions of distinctive mechano-biological stiffness. For example, mammary gland contains areas of soft adipose tissue, while the stromal/ductal areas are denser (Muschler and Streuli, 2010). In distinct individuals, the stroma contains regions of high or low mammographic density (Sherratt et al., 2016). This is due to patient-specific regions of the stroma having different biological stiffness (McConnell et al., 2016).

Matrix-secreting fibroblasts provide a model to understand peripheral circadian clocks (Balsalobre et al., 1998). Upon synchronization, these cells demonstrate remarkable circadian rhythms on stiff substrata, such as cell culture plastics, contrasting with weak clocks that we observed in epithelial cells on the same substrata. This suggests that fibroblast clocks differ from epithelia in terms of their response to ECM stiffness.

To reconcile these seemingly conflicting results, we investigated circadian clocks in primary epithelia and fibroblasts isolated from the same tissues. We discovered an inverse mechano-matrix control of epithelial and stromal circadian clocks in cells isolated from mammary gland, lung and skin. Thus, although cells originating from the epithelial and mesenchymal compartments of tissues contain circadian clocks, the role of the mechano-matrix environment in regulating clocks is opposite in these different cell types.

## RESULTS & DISCUSSION

### Mechano-sensitivity of mammary epithelial circadian clocks

Cell lines often have dramatically reduced tissue-specific functions, partly arising through extensive culture on non-physiological substrata. We, therefore, used primary cells isolated directly from mice, culturing them on substrata reflecting the stiffness of tissues *in vivo*.

**Fig. 1.**
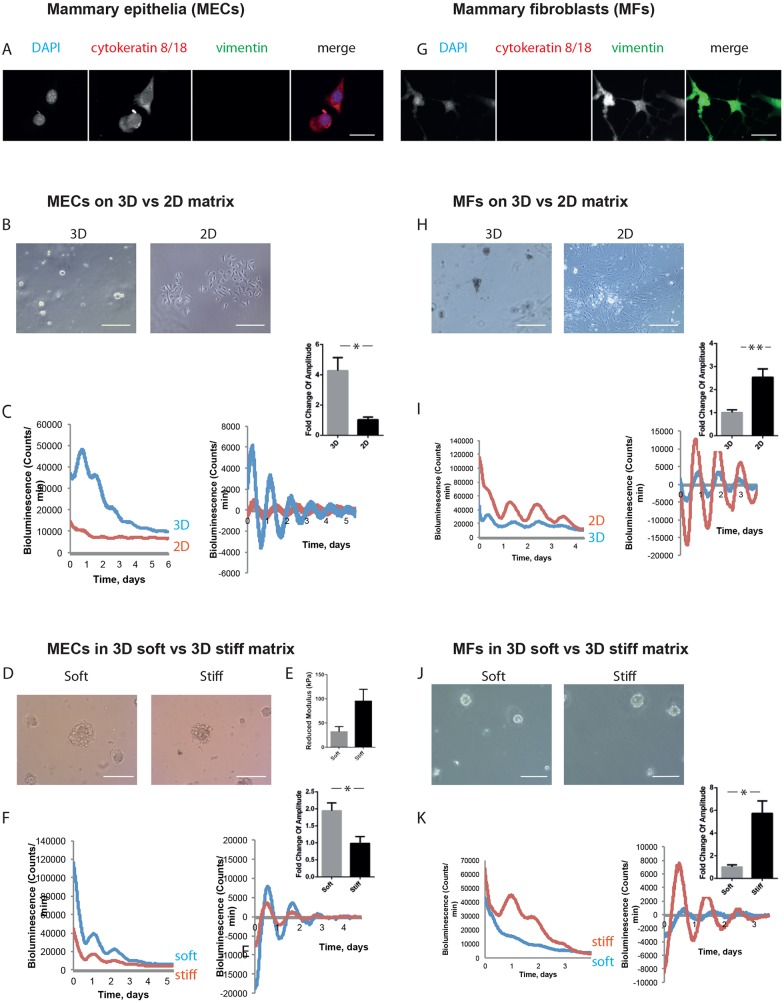
**Inverse mechano-sensitivity of mammary epithelial and fibroblast circadian clocks.** (A) Mammary epithelial cells (MECs) were isolated, and a pure epithelial population was confirmed by cytokeratin-positive and vimentin-negative staining. DAPI, blue; cytokeratin 8/18, red; vimentin, green (single-channel images shown in grey scale). Scale bar: 50 µm. (B) Phase-contrast images of MECs cultured in 3D and 2D. Scale bars: 50 µm. (C) MECs show larger amplitude of oscillation when cultured on 3D than 2D. Bioluminescence traces presented as raw (left) and normalised (right), with fold-change graph above. (Student's *t*-test, *P*<0.05, mean±s.e.m.). (D) Phase-contrast images of MECs cultured in soft and stiff alginate gels. Scale bar: 50µm. (E) Stiffness of soft and stiff gels, revealed by atomic force microscopy. (Student's *t*-test, *P*<0.05, mean±s.e.m.). (F) MECs show larger amplitude of oscillation when cultured in soft alginate gels rather than stiff gels. Bioluminescence traces presented as raw (left) and normalised (right), with fold-change graph above. (Student's *t*-test, *P*<0.05, mean±s.e.m.). (G) Mammary fibroblasts (MFs) were isolated by FACS. Immunofluorescence staining revealed MFs are cytokeratin-negative and vimentin-positive. DAPI, blue; cytokeratin 8/18, red; vimentin, green (single-channel images shown in grey scale). Scale bar: 50 µm. (H) Phase-contrast images of MFs cultured in 3D and 2D. Scale bars: 50 µm. (I) MFs show larger amplitude of oscillation when cultured on 2D plastic than 3D. Bioluminescence traces presented as raw (left) and normalised (right), with fold-change graph above. (Student's *t*-test, *P*<0.05, mean±s.e.m.). (J) Phase-contrast images of MFs cultured in soft and stiff alginate gels. Scale bar: 50 µm. (K) MFs show larger amplitude of oscillation when cultured in stiff alginate gels rather than soft gels. Bioluminescence traces presented as raw (left) and normalised (right), with fold-change graph above. (Student's *t*-test, *P*<0.05, mean±s.e.m.). For MECs, 3 independent biological replicates were performed, each with 2 animals, which were pooled together to form 2 cultures per condition. For MFs, 3 independent biological replicates were performed, each with 3 animals, which were pooled together to form 2 cultures per condition.

We obtained mammary epithelial cells (MECs) from non-pregnant glands of PER2::Luciferase clock reporter mice, which expressed epithelial-specific keratins (Fig. 1A) (Pullan and Streuli, 1996; Yoo et al., 2004). The cells were cultured on soft Matrigel in 3D or on collagen-coated stiff plastic dishes in 2D (Fig. 1B), followed by real-time bioluminescence photon counting using a LumiCycle. Under these conditions, the cells have dramatic differences in their ability to express tissue-specific genes, for example those encoding milk proteins (Streuli et al., 1991). Cells on 3D soft substrata showed high levels of synchronous circadian clocks (Fig. 1C). In contrast, those on 2D stiff substrata had weak clocks at levels that were several times lower than those in 3D-cultured cells.

To confirm that MEC clocks are controlled by microenvironmental stiffness, cells were cultured within both soft and stiff ECM 3D hydrogels under identical conditions apart from the gel's stiffness (Wood et al., 2017) (Fig. 1D). Atomic force microscopy revealed that the stiffness of the stiff gel (100 kPa) was 2.5-fold higher than the softer gel (Fig. 1E).

Cells formed clusters within the gels, where the extent of tissue-specific gene expression and cell function was dependent on ECM stiffness. By measuring the cycle of circadian clocks within MECs cultured in soft and stiff ECM, we found that the former showed considerably higher amplitude and magnitude of clock oscillations (Fig. 1F).

Thus, freshly isolated MECs express circadian clocks but they are under stringent mechano-matrix control.

### Mechano-sensitivity of mammary fibroblast clocks

We determined whether there is a similar mechano-matrix regulation of circadian clocks in stromal cells from the same tissue. Mammary fibroblasts were isolated from fresh tissue explants using FACS, being negatively sorted first for PECAM-1, Ly-51, Ter119 and CD45 (endothelial, immune, and red and white blood cells, respectively), and then for EpCAM and α6-integrin (epithelia). The resulting mammary fibroblasts were validated by confirming that they expressed vimentin but not keratin-containing intermediate filaments (Fig. 1G).

We examined the expression of circadian clocks in fibroblasts plated within Matrigel in 3D, or on plastic in 2D (Fig. 1H). In contrast to the epithelial cells, primary fibroblasts showed much stronger clocks when cultured on the stiffer substratum in 2D (Fig. 1I, Fig. S2, Movies 1,2). Similarly, the cells cultured within 3D hydrogels (Fig. 1J) showed much stronger clocks in the stiffer microenvironment than in the soft one (Fig. 1K).

Thus, regardless of mammary gland cell type, the strength of their circadian clocks is dependent on the mechanical stiffness of the ECM that they are in contact with. However, primary cultures of mammary epithelia and fibroblasts show opposite responses to the mechano-environment, with epithelial clocks being stronger in a soft matrix and fibroblastic clocks being strongest in stiff matrix. To the best of our knowledge, this is the first direct comparison of the epithelial and stromal clocks from the same tissue.

### Mechano-sensitivity of lung and epidermal clocks

**Fig. 2.**
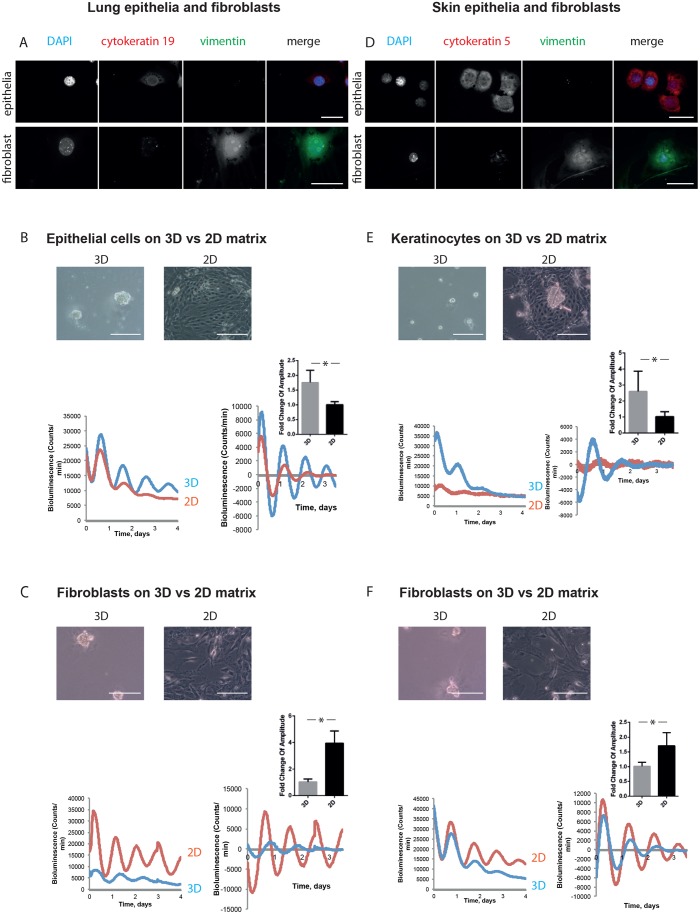
**Epithelia and fibroblasts from other tissues have inverse responses to mechanical stimuli.** (A) Immunofluorescence staining revealed lung epithelial cells as a population that is cytokeratin-positive and vimentin-negative. In contrast, lung fibroblasts are cytokeratin negative and vimentin positive. DAPI, blue; cytokeratin 19, red; vimentin, green (single-channel images shown in grey scale). Scale bar: 50 µm. (B) Lung epithelial cells have stronger circadian rhythms in 3D culture than 2D. Phase-contrast images above. Bioluminescence traces below, presented as raw (left) and normalised (right), with fold-change graph above. (Student's *t*-test, *P*<0.05, mean±s.e.m.). (C) Lung fibroblasts have weaker circadian rhythms in 3D culture than 2D. Phase-contrast images above. Bioluminescence traces below, presented as raw (left) and normalised (right), with fold-change graph above. (Student's *t*-test, *P*<0.05, mean±s.e.m.). (D) Keratinocyte isolation produces a population that is cytokeratin-positive and vimentin-negative. Dermal fibroblasts are a population that is cytokeratin-negative and vimentin-positive. DAPI, blue; cytokeratin 5, red; vimentin, green. Scale bar: 50 µm. (E) Keratinocytes have stronger circadian rhythms in 3D culture than 2D. Phase-contrast images above. Bioluminescence traces below, presented as raw (left) and normalised (right), with fold-change graph above. (Student's *t*-test, *P*<0.05, mean±s.e.m.). (F) Dermal fibroblasts have weaker circadian rhythms in 3D culture than 2D. Phase-contrast images above. Bioluminescence traces below, presented as raw (left) and normalised (right), with fold-change graph above. (Student's *t*-test, *P*<0.05, mean±s.e.m.). For lung epithelia, 3 independent biological replicates were performed, each with 3 animals, which were pooled together to form 2 cultures per condition. For lung fibroblasts, 3 independent biological replicates were performed, each with 2 animals, which were pooled together to form 2 cultures per condition. For keratinocytes, 3 independent biological replicates were performed, each with 5 animals, which were pooled together to form 2 cultures per condition. For dermal fibroblasts, 3 independent biological replicates were performed, each with 5 animals, which were pooled together to form 2 cultures per condition.

To determine whether this response is general, we examined primary cultures of cells from lungs and skin. For lungs, we isolated epithelia and fibroblasts by using established methods (Fig. 2A). When the epithelia from this tissue were plated onto a soft 3D ECM, their clocks showed high amplitude oscillations but were modest on stiff 2D ECM (Fig. 2B). The opposite profile of clock strength was seen in fibroblasts from the same tissue (Fig. 2C).

Studies with keratinocytes and the subtending dermal fibroblasts isolated from skin revealed a similar contrary ECM-dependence of clock strength (Fig. 2D-F). Higher amplitude (and magnitude in most cases) rhythms were observed in epithelia on softer 3D gels, while circadian rhythms in fibroblasts from the same tissue were stronger within a stiffer microenvironment.

Thus, epithelia have strong circadian clocks within a mechanically soft microenvironment, whereas stromal fibroblastic cells maintain robust clocks within a stiffer ECM-locale.

### Mechano-matrix control of cell type-specific expression of clock genes

To understand the link between mechanical properties of the ECM and the expression of clock genes, we first examined average expression levels in unsynchronised MECs cultured in soft and stiff matrices.

**Fig. 3.**
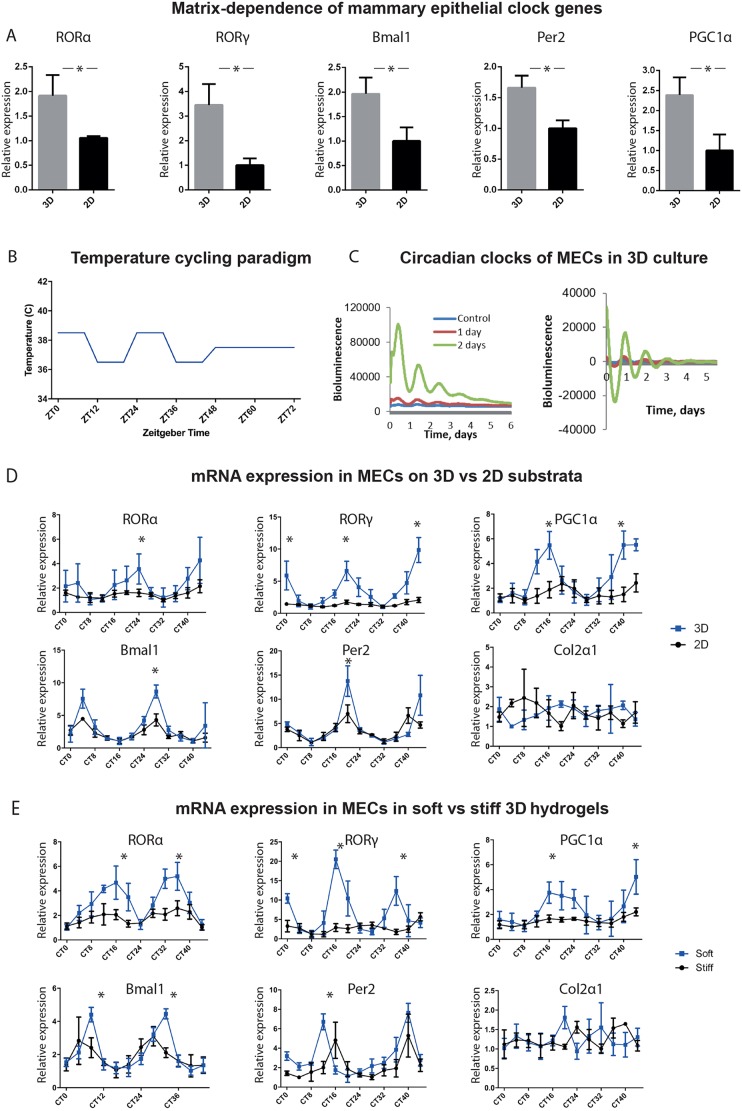
**Expression of clock genes is mechano-sensitive in MECs.** (A) Expression of genes encoding RORα, RORγ, Bmal1, Per2 and PGC1α is matrix-dependent, with higher levels of expression in MECs cultured in 3D vs 2D culture. *n*=3 biological replicates, each with 3 mice, pooled to form 3 cultures per condition, with a further 3 technical replicates for each gene. (Student's *t*-test, *P*<0.05 and *P*<0.01 for RORα; mean±s.e.m.). (B) Temperature cycling paradigm consists of 48 h in a temperature-controlled incubator, which cycles between 36.5°C and 38.5°C every 12 h, followed by 24 h at 37°C. For RT-qPCR, sampling begins thereafter (named as circadian time 0, CT0), every 4 hours until CT44. (C) MECs cultured in 3D show robust circadian oscillation that increases as the cells are in temperature cycles for longer times prior to recording. Bioluminescence traces presented as raw (left) and normalised (right). *n*=3 biological replicates, each with 4 mice, pooled to form 2 cultures per condition. (D) Time-course of clock and clock-controlled genes is significantly different in 3D vs 2D cultures. There is higher circadian mRNA expression in 3D for *RORα*, *RORγ*, *PGC1α*, *Bmal1* and *Per2* than in cells on 2D substrata. Note that there is no difference in the circadian expression of genes known not to be under circadian control in MECs, such as collagen2α1. *n*=3 biological replicates, each with 6 mice, pooled to form 36 cultures per condition. (Student's *t*-test, *P*<0.05, mean±s.e.m.). (E) Time-course of clock and clock-controlled genes is significantly different in cells in 3D soft versus stiff alginate gels. There is higher circadian expression in soft 3D for *RORα*, *RORγ*, *PGC1α*, *Bmal1* and *Per2*, than in cells in the stiff gels. *n*=3 biological replicates, each with 16 mice, pooled to form 36 cultures per condition. (Student's *t*-test, *P*<0.05, mean±s.e.m.).

Several clock genes, e.g. *ARNTL* (hereafter referred to as *Bmal1*), *Per2* and those expressing their regulators, e.g. *RORA* and *RORAC* (hereafter referred to as *RORα* and *RORγ*), and *PPARGC1A* (hereafter referred to as *PGC1α*), were mechano-matrix-dependent, with higher levels of expression in soft 3D ECM (Fig. 3A and Table S1). RORα, RORγ and PGC1α are nuclear hormone receptors that positively regulate *Bmal1* transcription (Canaple et al., 2003; Guillaumond et al., 2005; Yang et al., 2006).

Next, parallel MEC cultures were plated on Matrigel- and collagen-coated plates, or inside soft and stiff 3D alginate gels, and then entrained to have robust circadian timekeeping in a temperature-cycling incubator (36.5°C/38.5°C) for 48 h (Fig. 3B). This protocol maintains strong clock oscillations for several days after removing temperature cycles (Fig. 3C). RNA was extracted every 4 h for the subsequent 48 h, enabling circadian regulation of gene expression to be examined. A control gene, collagen 2a1 (Col2a1), showed no circadian regulation (Yang et al., 2017).

In Matrigel-cultured cells, there was a prominent increase in the amplitude and magnitude of the expression of several circadian genes, *RORα*, *RORγ*, *PGC1α*, *Bmal1* and *Per2*, (Fig. 3D). These genes were under much stronger circadian control when cultured in soft versus stiff 3D hydrogels (Fig. 3E). Of note, some other clock genes tested showed no change in response to altered matrix stiffness, i.e. *Per1*, *RORβ* (*RorB*), *Rev-erbβ* (*Nr1d2*), *Nfil3*, *TEF and Npas2*.

**Fig. 4.**
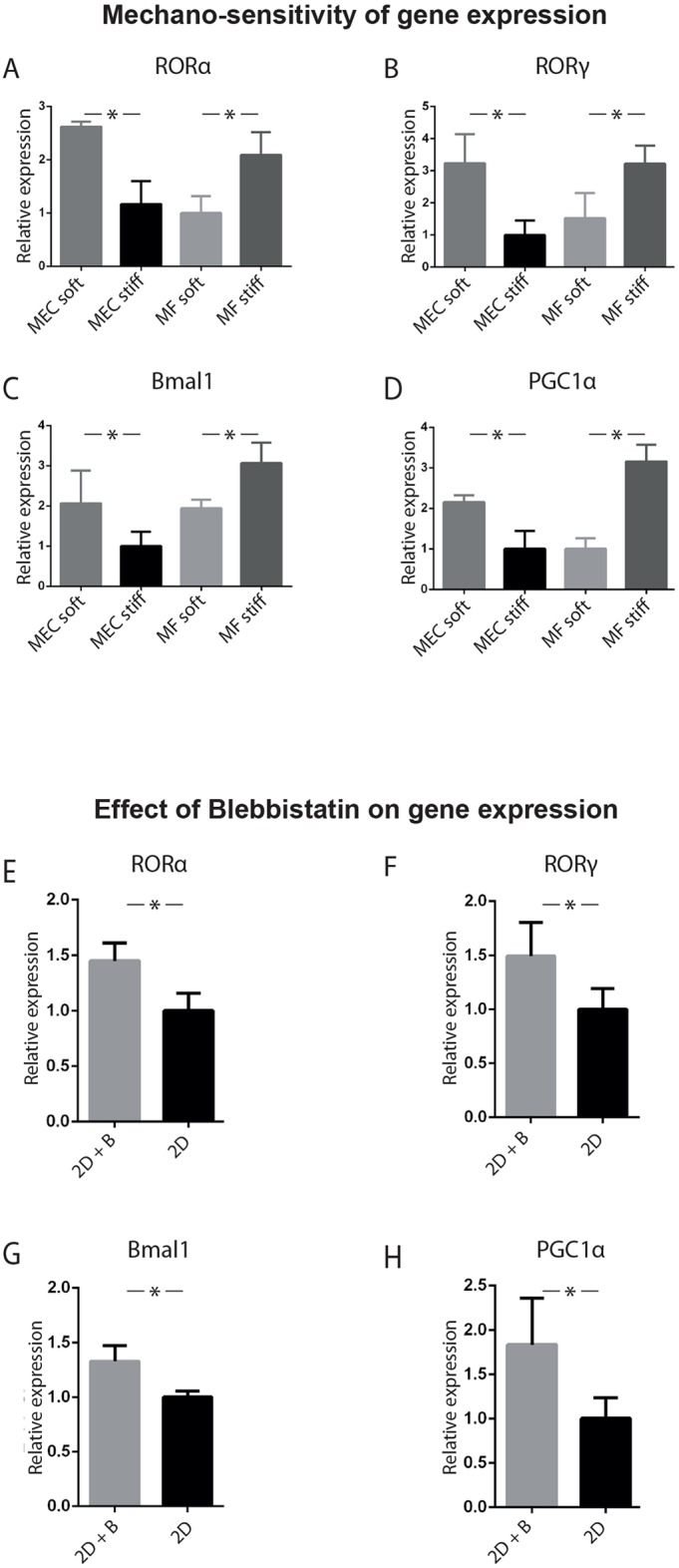
**Mechano-sensitivity of epithelial versus fibroblast gene expression.** (A-D) Validation of the changes in gene expression of (A) *RORα*, (B) *RORγ*, (C) *Bmal1*, (D) *PGC1α*. MECs and MFs were cultured in soft and stiff alginate gels. A single time-point RT-qPCR on unsynchronised cells revealed that in each case, there were higher levels of gene expression in MECs cultured in a soft ECM, and in MFs cultured in a stiff ECM. (One-way ANOVA, *P*<0.05, mean±s.e.m.). (E-H) Treating unsynchronised MECs in 2D for 2 h with 10 µM Blebbistatin (B), which is a Myosin-IIa inhibitor, increased the expression of clock genes, (E) *RORα*, (F) *RORγ*, (G) *Bmal1* and (H) *PGC1α*. (Student's *t*-test, *P*<0.05, mean±s.e.m.). For both experiments, *n*=3 biological replicates, each with 3 mice, pooled to form 3 cultures per condition.

To determine whether an inverse control occurred in fibroblasts, we examined expression profiles of their core clock genes. The expression of *RORα*, *RORγ*, *Bmal1* and *PGC1α* was significantly higher within a stiff mechano-environment (Fig. 4A-D).

Thus, the mechanism linking the mechano-physical nature of the ECM to circadian clocks occurs at the level of gene expression. Although there is mechano-matrix-dependent gene expression in both cell types, it occurs inversely in epithelia and fibroblasts.

### Clock mechano-sensitivity is mediated through the cytoskeleton

To explore how the mechano-matrix links to circadian clock gene expression, we examined the role of the actin-cytoskeleton (Yang et al., 2017). This structure acts as a mechanical link between the edge of the cell that contacts the ECM, which can be soft or stiff, and the nucleus. The cytoskeleton, thereby, provides communication between the cell exterior and transcriptional machinery.

MECs cultured on plastic were treated with the myosin-II inhibitor Blebbistatin, which suppresses cytoskeleton formation, reflecting the effect of plating cells in soft ECM. There was increased expression of clock genes, including *RORα*, *RORγ*, *Bmal1* and *PGC1α* (Fig. 4E-H), and this effect was less pronounced in fibroblasts (Fig. S3).

Thus, actin inhibition in MECs on stiff 2D substrata yields a similar outcome than plating cells on soft ECM, revealing that mechanical sensing of the microenvironment is mediated via the actin cytoskeleton.

### Conclusions

Our results reveal that circadian clocks are present within primary cultures of both epithelia and fibroblasts. Importantly, there is an inverse relationship between epithelial and fibroblast clocks in their responses to the mechano-matrix environment. Thus, in contrast to clocks in epithelial cells that favour softer matrix, fibroblasts prefer a stiffer matrix to maintain robust circadian rhythms.

Mechanistically, key regulators of the core clock gene *Bmal1*, including *RORα*, *RORγ* and *PGC1α*, were under mechanical control in MECs in a cell-matrix-dependent manner. The same genes were inversely regulated in fibroblasts.

The mechano-control of epithelial clocks is implicated in ageing and disease (Yang et al., 2017). Now we discovered an opposite mechano-matrix response in fibroblasts. Core circadian clock mechanisms are conserved among tissues, but outputs are cell-type and tissue-dependent. Disparities in the circadian phase of different cell types within the same tissue, such as in hair follicles, have been suggested (Janich et al., 2013). We now provide the first example of opposite clock responses to the same stimulus in epithelia versus fibroblasts.

Serum response factor (SRF) and myocardin-related transcription factor (MRTF) provide possible molecular links between stiffness and the clock. Indeed, the organisation of actin filaments changes throughout the day, driving the transcription of clock genes (Gerber et al., 2013). Activation of MRTF resets the circadian clock, in part by stimulating transcription of *Per2*, *RORα* and D site of albumin promoter binding protein (*Dbp*) (Esnault et al., 2014). Further work will determine whether the regulation of this pathway is conserved but opposite, between epithelia and fibroblasts.

Forthcoming studies in this area will lead to a deeper understanding of how clocks are mechanistically controlled by their local ECM niches. They may also reveal how dysregulated tissue mechanics alters homeostasis during ageing and in malignancy (Paszek et al., 2005; Sherratt et al., 2016). Interestingly, breast tissue stiffness varies amongst different individuals in the human population – those with a ‘stiffer’ stroma are more likely to develop breast cancer (Blakeman et al., 2016; McConnell et al., 2016). Understanding genome-wide clock targets in epithelia versus fibroblasts may help to determine cell-type specification of circadian clock functions.

## MATERIALS & METHODS

### Primary mammary epithelial and fibroblast cell culture

Primary MECs were purified from inguinal mammary glands of 2–3-month-old virgin mice and cultured in medium as described (Pullan and Streuli, 1996). Cells were stained and sorted using FACs, plated onto collagen-I-coated plastic Petri dishes for 2D monolayer cultures, basement membrane-matrix (Matrigel; BD Biosciences) to form 3D acini or in Alginate gels. Fibroblasts were cultured in DMEM containing 10% foetal calf serum (FCS) (Biowittaker), 50 U/ml penicillin/streptomycin, 0.25 mg/ml fungizone and 50 mg/ml gentamycin. For all bioluminescence recordings, the medium was changed and all the cells were cultured in the same medium.

### Fluorescence-activated cell sorting

Primary MECs were pelleted at 4350 rpm for 5′ and washed in Ca^2+^- and Mg^2+^-free PBS for 5′, treated with trypsin-DNase I solution (0.05% trypsin and 0.02% EDTA in Ca^2+^- and Mg^2+^-free PBS with 2000 U/ml DNase I, NEB DNase buffer and 1% penicillin/streptomycin) for 4′ at 37°C. 10% FCS was added, cells were washed, filtered through a 45-µm cell strainer, counted, antibodies were added, cells were stained for 45′, incubated for 45′ with antibodies against CD45 APC-Cy7, CD31-Biotin, Ter119-Biotin, BP-1-biotin, Streptavidin-APC-Cy7, CD326-APC and CD49f-eFluor450, washed and filtered through a 45-µm strainer into FACS-capped tubes (Table S2).

Biotin-conjugated antibodies were detected using Streptavidin-APC-Cy7. Cells were first sorted using APC-Cy7 into what are nominally called lineage-negative (Lin^−^) and lineage-positive (Lin^+^) cells. Lin^+^ cells include haematopoietic cells, endothelial cells and a proportion of the stromal compartment (largely immune cells) and were not used for subsequent sorts. Lin^−^ cells were sorted using CD326-APC and CD49f-eFlour450, producing three distinct populations, a CD326^low^ CD49f^low^ fibroblast population, a CD326^high^ CD49f^low^ luminal population, and a CD326^high^ CD49f^high^ basal population (Fig. S1, Movies 1,2).

### Lung epithelial cell culture

Murine trachea were dissected, digested overnight in 0.15% Pronase, washed, treated with DNase I, and plated onto collagen-I-coated plastic Petri dishes for 2D monolayer cultures or Matrigel (BD Biosciences) for 3D structures (Lam et al., 2011).

### Lung fibroblast cell culture

Primary lung fibroblasts were isolated from 2–3-month-old PER2::LUC mice (Meng et al., 2008b). Lungs were washed with ice-cold PBS, before being mechanically dissociated and enzymatically digested in a 100 U/ml collagenase IA solution at 37°C. Cells were then centrifuged, strained and plated on either 2D collagen-coated plastic or into Matrigel.

### Keratinocyte culture

Keratinocytes were harvested from PER2::LUC mice (Chacón-Martínez et al., 2017). Whole-skin isolates from the back of 6-day-old mice were digested in 0.08% trypsin for 1 h. Epidermis and dermis were then separated, and epidermal cells were strained through 45-µm cell strainers and pelleted at 900 rpm for 3′. For 2D monolayer culture, cells were cultured on fibronectin-coated plastic. For 3D culture, cells were cultured in Matrigel, with medium supplemented with 2% Matrigel.

### Dermal fibroblasts

Dermis and epidermis were separated; the dermis was minced into 1 mm^2^ pieces and incubated for 2 h in collagenase (400 U/ml), filtered through a 70 µm strainer, pelleted, resuspended and plated onto fibronectin-coated plastic or in Matrigel.

### Temperature synchronisation

Cells were synchronised using a programmable incubator. The program alternated between 38.5°C and 36.5°C every 12 h, for 48 h. After 48 h, the incubator temperature returns to 37°C for 24 h. At this point (named circadian time 0, CT0), cell harvesting for RT-qPCR begins. Cells were transferred from the incubator to the cell culture hood using a thermal plate at 37°C. Cells remained on the thermal plate until RNA extraction or being placed into a LumiCycle (Actimetrics) or photomultiplier devices (PMT).

### Bioluminescence recording

Tissues or cells cultured in 35-mm dishes were synchronised as described above. Prior to placing into the Lumicycle, normal culture medium was removed and rapidly replaced with pre-warmed HEPES- and sodium bicarbonate-buffered recording medium. The bioluminescence values for all cell types were recorded in this medium. Culture dishes were sealed with coverslips and vacuum grease, and placed into the LumiCycle or photomultiplier devices. Baseline subtraction was performed by using a 24-h moving average algorithm (Meng et al., 2008a; Dudek et al. 2018).

### RT-qPCR

Biological replicates (*n*=3), each with 16 mice, were pooled to form 36 cultures per condition. Cells were cultured in 12-well plates. At each time-point, 3 cultures per condition were used for RNA extraction with Qiagen RNeasy. cDNA was prepared using a High Capacity RNA-to-cDNA Kit (Applied Biosystems) and analysed for gene expression using quantitative real-time PCR (RT-qPCR) with TaqMan (Applied Biosystems) chemistry. RT-qPCR was performed in parallel with all biological replicates, and with two technical replicates per culture. Primer or probe mixes (Applied Biosystems) against the following genes were – *Per2*: Mm00478113_m1; *Bmal1* or *Arntl*: Mm00500226_m1; *Clock*: Mm00455950_m1; *Nr1d1* or *Rev-erba*: Mm00520708_m1; Cry1: Mm00514392_m1; *Dbp*: Mm01194021_m1; *RORα*: Mm00443103_m1; *RORγ*: Mm01261022_m1; *Bcar3*: Mm00600213_m1; *PrlR*: Mm04336676_m1; *Col2a1*: Mm01309565. Results were normalised to the values for *Gapdh* (Mm99999915_m1) expression, using the 2_ΔΔCt method (Livak and Schmittgen, 2001).

### Immunofluorescence

Indirect immunofluorescence was carried out on cells grown on ECM-coated coverslips. For fibroblasts, positive staining was for vimentin; for epithelial cultures, staining was for a specific cytokeratin. Cells were then imaged on a Zeiss Axioplan2 using a 63× / 1.40 Plan Apochromat objective and analysed with Axiovision v4.8.2 (Zeiss). Specific band pass filter sets for DAPI, FITC and Cy5 were used to prevent bleed through. Images were processed using Fiji ImageJ. Some data were generated with University of Manchester software; https://github.com/zindy/libatrous. Antibodies against the listed proteins were used as follows: Vimentin (diluted 1:1000, Santa Cruz, cat. no. sc-7557), pan-cytokeratin (diluted 1:1000, Abcam, cat. no. Ab27988), cytokeratin 5 (diluted 1:2000, Covance, cat. no. PRB-160P), cytokeratin 14 (diluted 1:1000, Covance, cat. no. PRB-155P), cytokeratin 8/18 (diluted 1:200, Progen, cat. no. Gp11) and cytokeratin 19 (diluted 1:10, generated in-house). Antibodies were assessed for specificity by western blotting. All antibodies detected bands only at the expected size.

### Atomic force microscopy

Whole alginate gels were mounted on glass slides and hydrated, then nano-indented with a spherically tipped cantilever (nominal radius 5 µm, spring constant 1 Nm^−1^, Windsor Scientific Ltd, Slough, UK) fitted to a Bioscope Catalyst AFM (Bruker, Coventry, UK) mounted on an Eclipse T1 inverted optical microscope (Nikon, Kingston, UK). Gels were indented 25 times over a 50 µm×50 µm area, with contact points evenly distributed across the area. Each gel was indented in 3 regions, and 3 gels were used per group. Force curves were analysed using Nanoscope Analysis v1.40 (Bruker). Curves were fit with a baseline correction before a force fit was applied to a Herzian (spherical) model with a maximum force fit of 70%. Contact-based values for reduced moduli were analysed using a Mann–Whitney U-test.

### Statistics and animal sampling

3-month-old virgin female 57BL/6J mice were used, sample size was determined by power analyses with an expected effect size of 33%, a common standard deviation of 15%, type I error rate of 0.05 and a desired power of 0.80. Exclusions were not applied. Tissues were pooled, cells were isolated, then split into experimental groups, effectively randomising the population. Appropriate statistical tests were devised by analysing the distribution and variance of the data.

## Supplementary Material

Supplementary information
