# Supplementary material for: Epithelial and stromal circadian clocks are inversely regulated by their mechano-matrix environment
Source: J Cell Sci. 2018 Mar 1;131(5):jcs208223. doi: 10.1242/jcs.208223 (PMC5897718; doi:10.1242/jcs.208223)
Supplement: Supplementary information [file joces-131-208223-s1.pdf]

Table S1. Gene expression in MEC cells cultured in different mechano-physical microenvironments.

GeneName	Description	P value	Log_2FC (3D Matrigel/ 2D plastic)
NM_008904	Mus musculus peroxisome proliferative activated receptor, gamma, coactivator 1 alpha (PGC1α), mRNA	0.0000	2.28
NM_011281	Mus musculus RAR-related orphan receptor gamma (RORγ), mRNA	0.0000	1.98
NM_011066	Mus musculus period homolog 2 (Drosophila) (Per2), mRNA	0.0000	1.76
NM_011146	Mus musculus peroxisome proliferator activated receptor gamma (Pparγ), mRNA	0.0000	1.74
AK041047	Mus musculus similar to Nuclear Receptor Subfamily 1, Group D, Member 1homolog (Nr1d1), mRNA	0.0084	1.22
NM_007771	Mus musculus cryptochrome 1 (photolyase-like) (Cry1), mRNA	0.0000	0.75
NM_013646	Mus musculus RAR-related orphan receptor alpha (RORα), mRNA	0.0007	0.61
NM_007489	Mus musculus aryl hydrocarbon receptor nuclear translocator-like (Arntl), mRNA	0.0044	0.54

Selected clock-related genes from a set of upregulated genes in unsynchronized primary MECs cultured in Matrigel compared to plastic. Data were obtained from Agilent mouse oligo microarray, and are displayed as log2 fold change of expression and significance value.

Table S2: Antibodies used in this study.

Antibody	Recognises	Tag	Dilution	Supplier	Cat Number
Anti-CD45	Ly-5	APC-Cy7	5µl/million cells	BD Pharmigen	557659
Anti-CD31	PECAM-1	Biotin	5µl/million cells	BD Pharmigen	558737
Anti-Ter-119	Ly-76	Biotin	5µl/million cells	BD Pharmigen	553672
Anti-BP-1	Ly-51	Biotin	5µl/million cells	BD Pharmigen	553159
Streptavidin	Biotin	APC-Cy7	5µl/million cells	BD Pharmigen	554063
Anti-CD326	EpCAM	APC	5µl/million cells	eBioscience	17-5791
CD49f	Integrin α 6	eFluor450	5µl/million cells	eBioscience	48-0495

Journal of Cell Science • Supplementary information

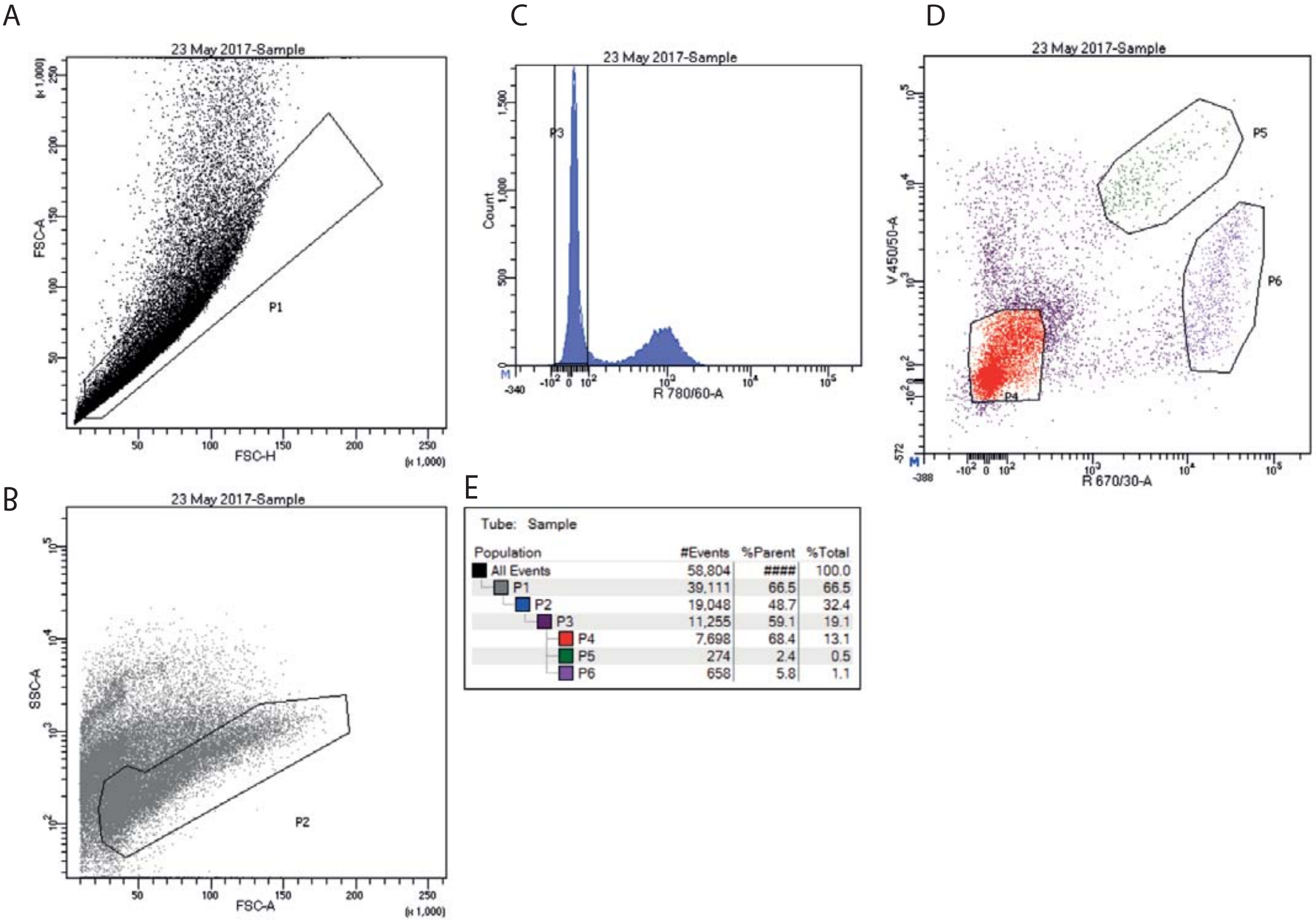

Population	#Events	%Parent	%Total
All Events	58,804	####	100.0
P1	39,111	66.5	66.5
P2	19,048	48.7	32.4
P3	11,255	59.1	19.1
P4	7,698	68.4	13.1
P5	274	2.4	0.5
P6	658	5.8	1.1

Fig. S1. Sorting cascade for isolating mammary fibroblasts.

Live, single cells were negatively sorted against a cocktail of antibodies to remove endothelial, immune and hematopoietic cells. Negative cells were then sorted against EpCAM and CD49f. CD49f- and EpCAM-low cells form the putative mammary fibroblast population.

A-B) Forward and side scatter plots with gates set to pull out single, live cells.

C) Staining with lineage cocktail reveals an unstained population of fibroblasts and epithelia.

D) Mammary fibroblasts stain negatively for CD49f and EpCAM.

E) Number of cells within a population expressed as a percentage of their parent population and total cells.

Journal of Cell Science • Supplementary information

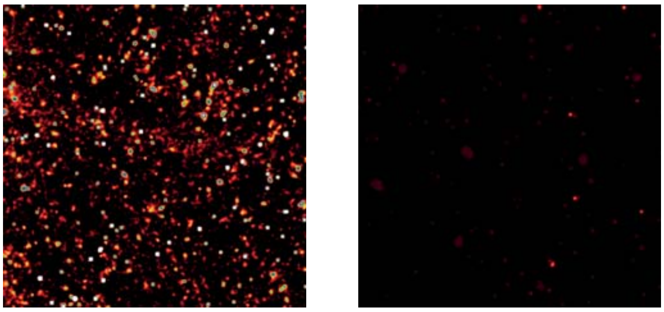

Fig. S2. Stiffness-dependent circadian clocks in mammary fibroblasts.
Bioluminescence imaging of PER2::Luc oscillation of mammary fibroblasts cultured under 2D (left) and 3D (right) conditions (see also Movie 1 and Movie 2, respectively). Shown are stills of Movie 1 and 2, with the images at brightest luminescence. The movies were reanalysed using software generated by Dr Egor Zindy at the University of Manchester (https://github.com/zindy/libatrous), and displayed using false colours (ImageJ "iman" look-up table).

Journal of Cell Science • Supplementary information

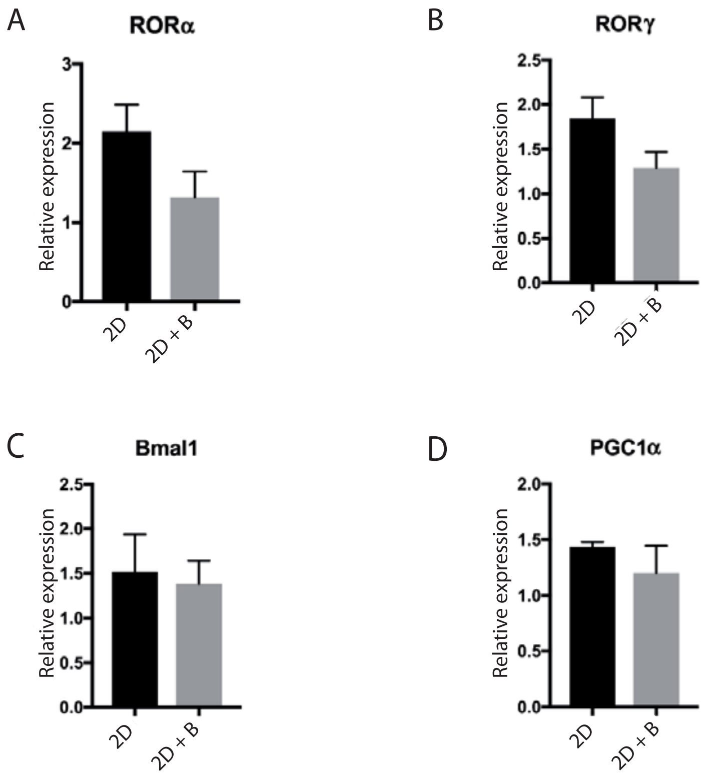

Fig. S3. Mechano-response of circadian clock genes mediated by the actin cytoskeleton in mammary fibroblasts.

A-D) Treating unsynchronised MFs in 2D for 2 hours with 10 µM Blebbistatin:

A) RORα

B) RORγ

C) Bmal1

D) PGC1α

(Student's t-test, $p<0.05$, Mean+s.e.m.)

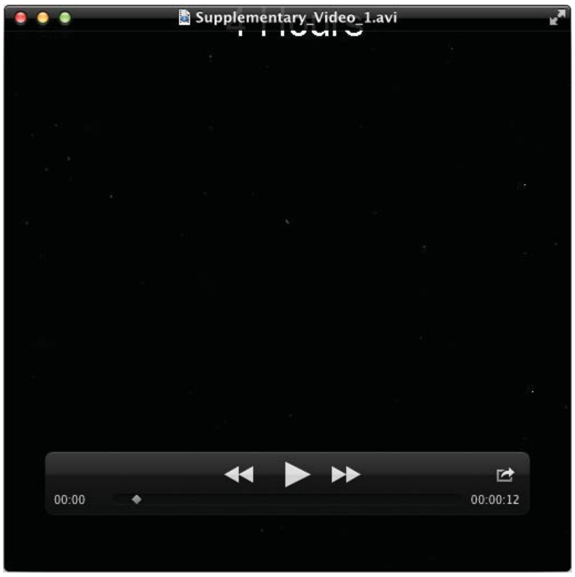

Movie 1. Stiffness-dependent circadian clocks in mammary fibroblasts; fibroblasts cultured in 2D (see Fig. S2).

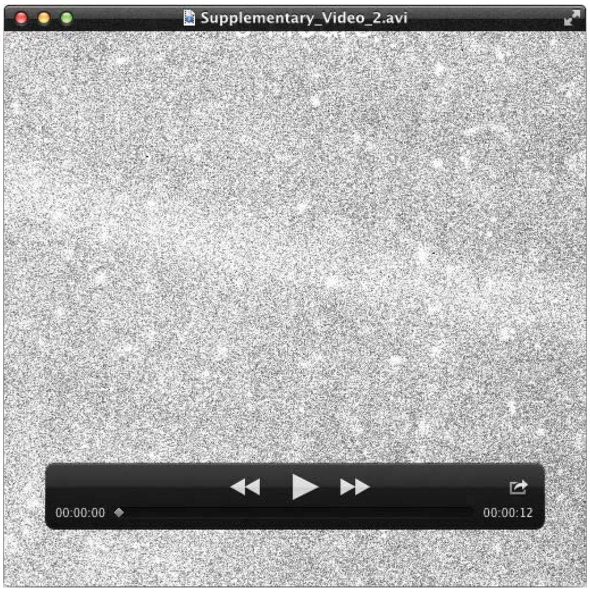

Movie 2. Stiffness-dependent circadian clocks in mammary fibroblasts; fibroblasts cultured in 3D (see Fig. S2).

Real-time bioluminescence imaging of PER2::Luc oscillation of mammary fibroblasts cultured under 2D (Movie 1) and 3D (Movie 2) conditions. Due to the weak bioluminescence emission in 3D, the images were reanalysed in Fig S2.
